# Ultrafast biexciton spectroscopy in semiconductor quantum dots: evidence for early emergence of multiple-exciton generation

**DOI:** 10.1038/srep03206

**Published:** 2013-11-13

**Authors:** Younghwan Choi, Sangwan Sim, Seong Chu Lim, Young Hee Lee, Hyunyong Choi

**Affiliations:** 1 School of Electrical and Electronic Engineering, Yonsei University, Seoul 120-749, Republic of Korea; 2 IBS Center for Integrated Nanostructure Physics, Institute for Basic Science (IBS), Daejon 305-701, Republic of Korea; 3 Department of Energy Science, Sungkyunkwan University, Suwon, 440-746, Republic of Korea

## Abstract

Understanding multiple-exciton generation (MEG) in quantum dots (QDs) requires in-depth measurements of transient exciton dynamics. Because MEG typically faces competing ultrafast energy-loss intra-band relaxation, it is of central importance to investigate the emerging time-scale of the MEG kinetics. Here, we present ultrafast spectroscopic measurements of the MEG in PbS QDs via probing the ground-state biexciton transients. Specifically, we directly compare the biexciton spectra with the single-exciton ones before and after the intra-band relaxation. Early emergence of MEG is evidenced by observing transient Stark shift and quasi-instantaneous linewidth broadening, both of which take place before the intra-band relaxation. Photon-density-dependent study shows that the broadened biexciton linewidth strongly depends on the MEG-induced extra-exciton generation. Long after the intra-band relaxation, the biexciton broadening is small and the single-exciton state filling is dominant.

The limiting factor for improving solar-cell efficiency lies in the simple physics that single-photon absorption generates one electron-hole pair^1^. The possibility of generating multiple charge carriers per photon, known as carrier multiplication (CM) or multiple exciton generation (MEG), is of crucial importance for developing efficient solar-cell devices^2^,^3^,^4^,^5^,^6^,^7^,^8^. Semiconductor quantum dots (QDs) represent well-defined structures to explore the quantum limit of harnessing solar-conversion efficiency^9^,^10^,^11^,^12^,^13^. By engineering the sizes of QD composites, it has been demonstrated that not only the optical properties^14^,^15^, but also the MEG efficiency in QDs can be modified^16^. MEG in a photo-excited QD system is a prominent route for enhancing the conversion efficiency because carriers confined in spatial dimensions that are smaller than the bulk exciton Bohr radius lead to the formation of discrete excitonic states such that efficient MEG is possible either by suppressing the ultrafast electron-phonon relaxation^4^,^17^,^18^ or by enhancing the Coulomb interactions via reduced dielectric screening at the QD surface^19^.

Numerous investigations have shown that the kinetic origin of MEG dynamics in QDs is intrinsically complex because the photo-generated single exciton initially suffers from extremely fast intra-band relaxation^20^,^21^,^22^, whose interaction time-scale is typically in the range of a few ps^6^. To enhance the MEG efficiency, it is desirable to circumvent the ultrafast energy-loss intra-band process^23^,^24^. Recent studies suggest that the MEG is an instantaneous phenomenon occurring before the intra-band energy relaxation^25^ via virtual single excitonic^26^ or biexcitonic optical transition^27^ or coherent superposition among multi-exciton states^12^. Other investigation suggests that the intra-band relaxation rate competes with the MEG formation rate^6^.

The above mentioned photo-physical complexity of MEG is largely due to the nature of intrinsic multi-particle (or multi-exciton) interaction^28^. When more than two excitons are created under high-energy excitation condition, the lowest lying energy state is not the single exciton; the mutual interaction between two excitons results in the formation of a Coulomb-correlated two excitonic state, called biexciton^29^,^30^,^31^,^32^,^33^. The biexciton is energetically more stable than the single exciton such that it exists below the single-exciton state^32^,^34^. Recent studies have reported that the final biexciton density strongly influences the solar-conversion efficiency^25^,^26^,^35^. Although it is important to study the impact of the MEG on the transient biexciton spectra, no experimental investigations have been provided to compare the MEG-induced biexciton dynamics with the intra-band relaxation dynamics.

The key experimental observation in this study is that the optically-induced MEG is an extremely fast process, arising before the intra-band relaxation. By exploring the lowest observable biexciton dynamics, we directly measure that the biexciton bleaching comes from early emergence of the photo-induced MEG, in which the effect of extra-exciton generation is manifested by the increased broadening of the biexciton linewidth via multi-exciton interaction. Note that, in contrast to the conventional single-exciton MEG spectroscopy^11^,^36^,^37^,^38^,^39^, our ultrafast time-resolved experiments were performed both in the MEG and in the non-MEG regimes via photon-energy and density-controlled measurements on the single- and biexciton spectra.

## Results

### Single-exciton MEG dynamics

**Figure 1:**
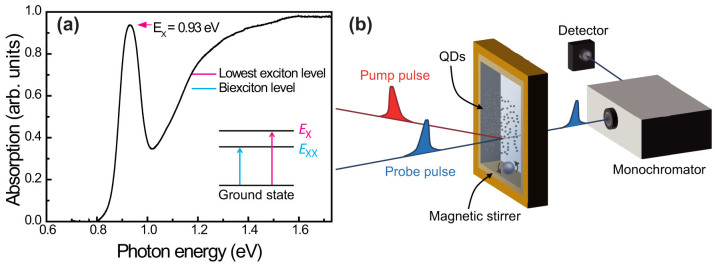
QD absorption spectra and experimental setup. (a) Linear absorption spectra of the colloidal PbS QDs used in the study. Inset: schematic energy levels for the single-exciton *E*_x_ and the ground-state biexciton *E*_xx_, respectively. (b) Schematic for the ultrafast pump-probe measurements. For the spectrally-resolved measurements, the probe pulse is scanned through a monochromator (Newport 74125 Oriel Cornerstone 260 1/4 m) at each Δ*t*. The measured FWHM of the pump and probe beam are 150 *μ*m and 100 *μ*m, respectively. All measurements are performed at room temperature.

Figure 1a shows data for the broadband optical absorption of the colloidal semiconductor PbS QDs and Fig. 1b shows a schematic for the ultrafast pump-probe measurements (See method for the detailed description of sample preparation and ultrafast spectroscopy). The lowest single-exciton bandgap energy *E*_x_ is identified as 0.93 ± 0.01 eV, and the ground-state biexciton energy *E*_xx_ is estimated to be 0.87 ± 0.03 eV^30^,^31^,^32^,^33^.

Before the discussion on the biexciton dynamic, it is instructive to present detailed measurements on the intra-band relaxation dynamics because the linewidth broadening of single excitons and biexcitons is necessary related to the competing relaxation rate between the MEG and the intra-band dynamics, in which the time scale of the intra-band relaxation is typically a few ps^16^,^40^,^41^, comparable with the MEG time scale. In the experiment, the colloidal semiconductor PbS QD sample was pumped by two different pump-photon energy *E*_pump_ with 1.55 eV and 3.10 eV, and the average number of initially photo-generated excitons per QD 〈*N*_0_〉, or initial exciton occupancy, was controlled from 0.1 to 2.2 to investigate the photon density-dependent *E*_x_ dynamics.

**Figure 2:**
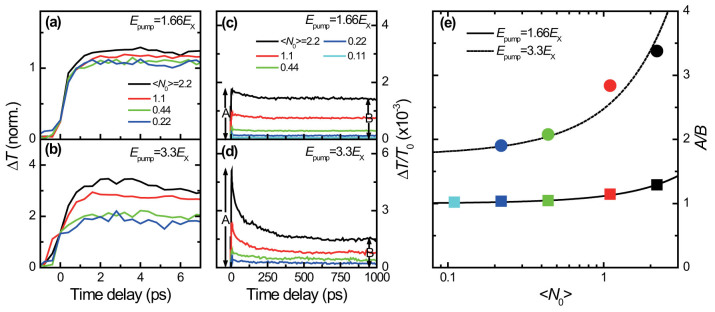
Single-exciton MEG dynamics. Single-exciton dynamics in a short pump-probe range between −1 and 7 ps for 1.66*E*_x_ (a) and 3.3*E*_x_ excitations (b). The differential transmission signals probed at *E*_x_ with the 1.66*E*_x_ pump (c) and the 3.3*E*_x_ pump (d) are displayed with 〈*N*_0_〉 ranging from 0.11 to 2.2. Here, 〈*N*_0_〉 is estimated via 〈*N*_0_〉 = *j_p_σ_a_*, where *j_p_* is the pump fluence in unit of photons per cm^2^ and *σ_a_* is the absorption cross-section in unit of cm^2^ 14. (c) The experimentally measured *A/B* ratio is shown for the 1.66*E*_x_ pump (filled circle) and for the 3.3*E*_x_ pump (filled square). The calculated *A/B* ratio is obtained via Poisson distribution of the QD occupancies for the 1.66*E*_x_ pump (solid line) and for the 3.3*E*_x_ pump (dashed line).

In order to determine the intra-band relaxation rate, we measured the *E*_x_ dynamics in a short Δ*t* range between −1 ps and 7 ps as shown in Figs. 2a and b. By examining the rising edge of the *E*_x_ peak, we show that the relaxation process is completed at pump-probe delay Δ*t* = 1 ps for 1.66*E*_x_ excitation (non-MEG regime) and Δ*t* = 2 ps for 3.3*E*_x_ excitation (MEG regime). This 2 ps time constant is consistent with prior experimental studies of hot-carrier MEG dynamics in PbS quantum dots, where the reported value of intra-band relaxation is in the range of 2–2.5 ps^16^,^40^,^41^.

Figure 2c shows the *E*_x_ transients excited by low *E*_pump_ ( = 1.66*E*_x_). The observed step-like signals with a small *A*/*B* ratio (amplitude ratio of the early to late pump-probe delay Δ*t*) are not attributed to the MEG transients, because the MEG typically requires *E*_pump_ greater than a few *E*_x_. When the QDs are excited by high *E*_pump_ ( = 3.3*E*_x_), we observed fast (90 ps) and slow decay (~100 ns) components with a large *A*/*B* ratio, as depicted in Fig. 2d. The experimentally determined *A*/*B* ratio of the QD occupancy was modelled via Poisson statistics (Fig. 2e)^42^. Since multiple excitons generated by the MEG decay via Auger recombination, the amplitude at long Δ*t* (denoted by *B* in Fig. 2c and d) provides a scaling factor for calculating the exciton multiplicity 〈*N_x_*〉 = *A*/*B*, where *A* is the amplitude of single-exciton population immediately after pump excitation (denoted by *A* in Fig. 2c and d). By comparing the measured *A*/*B* ratios in the limit of 〈*N*_0_〉 → 0, a strong indication of the MEG for the 3.3*E*_x_ pump was identified^43^. As reported previously^16^,^36^,^38^,^40^,^44^, these observations confirm that the typical MEG dynamics are observable via probing the *E*_x_ dynamics.

### Transient Stark shift and biexciton linewidth broadening

**Figure 3:**
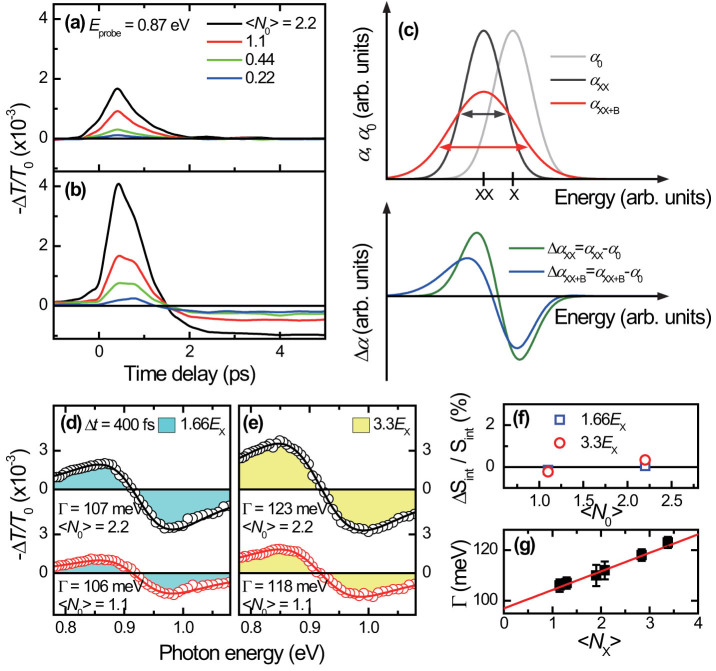
Ultrafast MEG-induced biexciton transients before the intra-band relaxation. Transient *E*_xx_ dynamics are shown as a function of Δ*t* for the 1.66*E*_x_ pump (a) and for the 3.3*E*_x_ pump (b) with various 〈*N*_0_〉. (c) Schematic illustration of the PA caused by the transient Stark shift of the single-exciton absorption (black line) and the corresponding photo-induced biexciton broadening (red line). The gray line indicates the single-exciton absorption without the pump. The differential PA spectra exhibit an energy-shifted broader feature (blue line) due to the MEG-induced exciton scattering compared to the case of no MEG (green line). The spectrally-resolved PA spectra with the probe range from 0.78 to 1.08 eV measured at Δ*t* = 400 fs are shown for the 1.66*E*_x_ pump (d) and for the 3.3*E*_x_ pump (e) with two different 〈*N*_0_〉. Solid lines represent numerical fits using equation (1). The obtained biexciton Γ for the 1.66*E*_x_ pump with 〈*N*_0_〉 = 1.1 and 2.2 are 106 meV and 107 meV, respectively, and those for the 3.3*E*_x_ pump with 〈*N*_0_〉 = 1.1 and 2.2 are 118 meV and 123 meV, respectively. (f) Spectrally integrated areas of the broadened biexciton absorption. (g) The biexciton Γ broadening linearly increases with increasing the total number of excitons. The experimentally determined Γ (black filled squares) from the Figs. 3d and e are compared to the equation (2).

The central issue to address in this paper is to investigate how the biexciton dynamics is influenced by the early formation of MEG. Figures 3a and b display the biexciton transients for the 1.66*E*_x_ pump and 3.3*E*_x_ pump as a function of Δ*t* with controlled excitations from 〈*N*_0_〉 = 0.22 to 〈*N*_0_〉 = 2.2. Immediately after pump excitation, the photo-induced absorption (PA) exhibits rapid bleaching at *E*_xx_ within the first Δ*t* = 400 fs with a much larger PA peak for the 3.3*E*_x_ pump than the 1.66*E*_x_ pump. While both signals decay non-exponentially, the signals pumped by 1.66*E*_x_ decay to zero after a few ps, and the transients pumped by 3.3*E*_x_ change their signs from positive to negative near Δ*t* = 2 ps.

In a strong quantum-confinement regime, the pump-created local electric field induces a large transient shift of absorption, a phenomenon known as transient Stark shift^42^,^45^. This effect is more considerable with increasing photo-generated carriers, which in turn produces a stronger local field and complicates the ultrafast PA spectra as schematically shown in Fig. 3c. Note that the increased carrier density is reflected both by the carrier-induced Stark shift and by the absorption linewidth Γ that leads to a broader feature^46^,^47^. As discussed later, this broadened Γ directly determines the effect of MEG on the biexciton dynamics through extra-exciton generation.

It is expected that high *E*_pump_ excitation, larger than *E*_x_, enhances the Γ broadening due to the extra-exciton generation. Immediately after the pump (Δ*t* = 400 fs), we clearly observe that the biexciton Γ is broader for the 3.3*E*_x_ excitation case than for the 1.66*E*_x_ one, as shown in Figs. 3d and e with two different excitations of 〈*N*_0_〉 for each *E*_pump_ excitation. Thus, the observed transient PA dynamics can be understood by combined effects of both the carrier-induced transient Stark shift and the MEG-induced biexciton Γ broadening. We additionally notice that the spectrally-integrated areas of the broadened biexciton absorption remain the same regardless of 〈*N*_0_〉 as shown Fig. 3f. This constraint indicates that the broadening is determined by the number of excitons, and it ensures that the biexciton PA peak is reduced by the exciton-exciton collision-induced broadening rather than the phase-space filling argument^46^.

### Quantitative analysis of the MEG-induced biexciton broadening and the early emergence of MEG

The entire pump-induced changes of the absorption spectra can be faithfully fit via the following third-order susceptibility function^31^,^33^, 
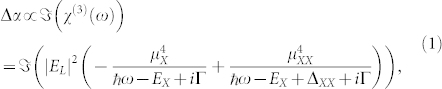
where *E_L_* is the electric field of the pump, Δ*_XX_* is the biexciton binding energy, and *μ_X_* and *μ_XX_* are the transition dipole moments from the ground state to *E*_x_ and to *E*_xx_, respectively. The first term represents the bleaching at *E*_x_ and the second term represents the PA at ground-state *E*_xx_. For the PA dynamics measured at Δ*t* = 400 fs (Figs. 3d and e), because the intra-band relaxation time (2 ps) is longer than Δ*t* of 400 fs, the absorption change measured at *E*_x_ was not induced by the single-exciton state filling. In addition, Auger recombination and impact ionization (Auger processes) can be neglected because the time-scale of Auger processes is much slower (100 ~ 200 ps) than the intra-band relaxation. On the other hand, the difference in Γ, obtained from a fit of equation (1) to the measured PA spectra, shows that the broadening is associated with the MEG-induced biexciton broadening.

For quantitative analysis, the biexciton Γ is plotted as a function of the average number of total excitons per QD 〈*N_x_*〉, and the results are displayed in Fig. 3g. Here, we note that the definition of 〈*N_x_*〉 (obtained from the measured *A*/*B* ratios in Fig. 2c) differs from that of 〈*N*_0_〉 in a sense that 〈*N_x_*〉 includes both the average number of initially photo-generated excitons and the MEG-induced excitons per QD; 〈*N*_0_〉 is the average number of photo-generated exciton per QD^11^. In other words, the biexciton broadening is directly related to the total number of excitons 〈*N_x_*〉, not by the initial exciton occupancy 〈*N*_0_〉. By plotting the Γ as a function of 〈*N_x_*〉, we obtain a linear relationship of 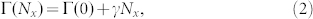
where *γ* ( = 6.8 meV per exciton) is the Γ broadening parameter per exciton. Because Γ(0) represents the linewidth broadening in the absence of photo-generated excitons, the value should corresponds to the *E*_x_ broadening in Fig. 1a. A simple Gaussian fit shows that the *E*_x_ broadening in Fig. 1a is 100 ± 5 meV, well corroborated with the fitted Γ(0) = 98 meV of the biexciton broadening. The characteristic broadening of Γ with increasing 〈*N_x_*〉 entails the effect of MEG, i.e. as more excitons are injected, more broaden feature of biexciton Γ is expected.

## Discussion

**Figure 4:**
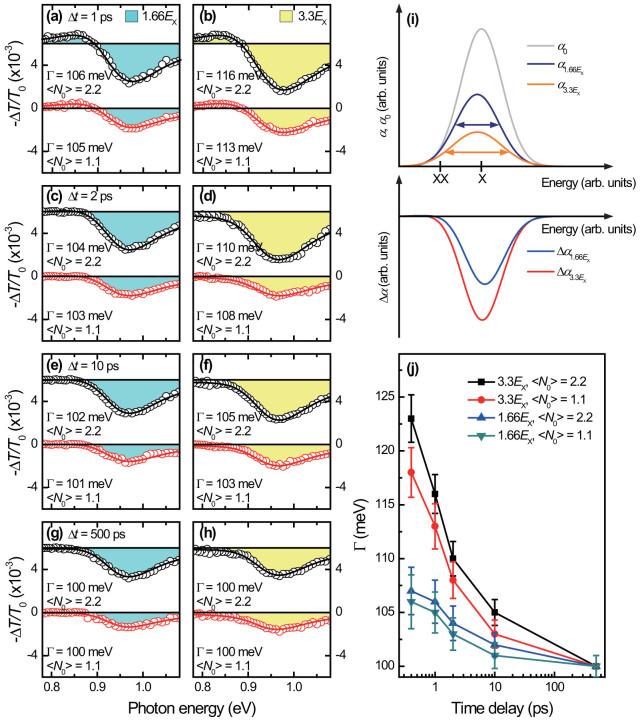
Biexciton broadening at longer delays than 400 fs. The single- and biexciton absorption change spectra at Δ*t* of 1 ps (a,b), 2 ps (c,d), 10 ps (e,f) and 500 ps (g,h) with two *E*_pump_. (i) Schematic illustration of the PA bleaching dynamics at longer Δ*t* > 2 ps for the 1.66*E*_x_ pump (blue line) and for the 3.3*E*_x_ pump (orange line). (j) Transient Γ broadening is shown as function of Δ*t*.

The early emergence of the MEG is substantiated by measuring the single- and biexciton spectra before/after the intra-band relaxation of 2 ps. It is expected that Γ should be large if Δ*t* is shorter than the intra-band relaxation time, i.e. if the MEG-induces exciton-exciton scattering occurs earlier than the intra-band relaxation, Γ before the intra-band relaxation is larger than Γ after intra-band relaxation. Figures 4 (a) and (b) show the PA signals at Δ*t* = 1 ps. As expected, the Γ broadening at Δ*t* = 1 ps is smaller than at Δ*t* = 400 fs, but larger than at Δ*t* = 2 ps. Figures 4c and d show the spectra at Δ*t* = 2 ps for the 1.66*E*_x_ pump and for the 3.3*E*_x_ pump, respectively. The Γ at 2 ps for 3.3*E*_x_ with 〈*N*_0_〉 = 2.2 is 110 meV while the Γ at Δ*t* = 400 fs with same condition is 123 meV. Indeed, we clearly see that Γ at Δ*t* = 2 ps is smaller than that of before intra-band relaxation both for the two 〈*N*_0_〉 excitations (see Fig. 3e and Figs. 4b and d).

Long after the intra-band relaxation finishes, the carrier-induced Stark shift becomes weak, and the single-exciton state filling is dominant (Figs. 4e–h). As schematically shown in Fig. 4i, the weak Stark shift is rendered as the absence of PA signals at 0.85 eV, but the effect is not completely vanished; negative PA peaks appear at 0.97 eV instead of the single-exciton energy of 0.93 eV in Fig. 1a. Because the PA peak is proportional to the generated exciton numbers, the magnitude of bleaching is larger for the case of 3.3*E*_x_ pump than the 1.66*E*_x_ pump case. We note that the chosen two *E*_pump_ (1.66*E*_x_ and 3.3*E*_x_) set the below and upper limit on the occurrence of MEG such that the observed two dynamics (before and after the intra-band relaxation) are distinguishable in comparing the MEG-induced biexciton lineshape and the single-exciton-dominated one. There is a possibility that significant re-shaping of single-exciton spectra can be observed at longer Δ*t*, which may occur when as many as 50% of QDs are occupied by multiple electron-hole pairs (i.e. 〈*N*_0_〉 < 1). This scenario can be excluded in our investigation because the PA peaks at Δ*t* = 2 ps show negligible energy shifts^48^ even when 〈*N*_0_〉 > 1.

To investigate the effect of Auger and single-exciton recombination on Γ, we compare the PA spectra at Δ*t* of 10 ps and 500 ps. We noted that the single-exciton decay dynamics consists of two relaxation components (see Figs. 2c and d): one is “fast” Auger recombination (known as biexcitonic relaxation component^6^) and another is “slow” single-exciton recombination (referred to as excitonic background^6^). Figures 4e and f display the PA spectra at Δ*t* = 10 ps. Because the Auger recombination is not completed, Γ at Δ*t* = 10 ps is smaller than Γ at Δ*t* = 2 ps. After the Auger recombination is finished, 〈*N_x_*〉 at Δ*t* = 500 ps approaches one both for the 1.66*E*_x_ and 3.3*E*_x_ pump cases. Because nearly one exciton is left at Δ*t* = 500 ps, Γ for both *E*_pump_ (Figs. 4g and h) is identical with Γ of 100 meV, representing negligible effect of single-exciton recombination on Γ.

The measured data are summarized in Fig. 4j. Two main aspects are addressed. First, Γ at Δ*t* = 400 fs is the largest compared to the Γ at Δ*t* > 400 fs, providing an evidence for the large biexciton Γ broadening in early Δ*t*. Second, by observing the fact that the decreasing slope of Γ with Δ*t* for 3.33*E*_x_ excitation is steeper than the 1.66*E*_x_ excitation up to Δ*t* = 2 ps, we can find that the effect of MEG on Γ is strongly influenced by extra-exciton generation before the intra-band relaxation.

To conclude, we have investigated the transient dynamics of biexciton, located below the single-exciton energy, and have explored the impact of MEG on the biexciton spectra. Our ultrafast spectroscopy shows that the linewidth broadening of the biexciton spectra provides direct evidence on the early emergence of the MEG compared to the intra-band relaxation time. We additionally have presented quantitative analysis that the broadening parameter Γ per exciton increases linearly with increasing the total number of excitons. For detailed time-resolved spectral analysis, the PA spectra are compared with single-exciton ones at Δ*t* = 400 fs and longer delays. The comparison underscores that Γ broadening before Δ*t* = 2 ps is larger than the Γ after Δ*t* = 2 ps, corroborating that the MEG indeed occurs before the intra-band relaxation.

## Methods

### Synthesis of PbS quantum dots

Our PbS colloidal quantum dots are capped using eleic acid and dispersed in toluene. The synthesis of the sample followed a procedure that used standard air-free solution based technique^49^. In a typical synthesis, 2.0 mmol of PbO (0.445 g), 8.0 mmol (2.25 g) of oleic acid (OA), and 9.9 mmol (2.5 g) of 1-octadecene (ODE) are placed in a flask and heated to 100°C under vacuum, and then nitrogen was introduced. The temperature was controlled to the appropriate injection temperature (100 to 150°C) to obtain the desired particle size. The sulfur precursor was prepared by mixing bis(trimethylsilyl)sulfide with ODE. Removal of excess ligand was completed by repeated the followings: precipitation in acetone, centrifugation of the particles, and dispersion in toluene.

### PbS QDs and ultrafast spectroscopy

The sample used in this experiment is semiconductor colloidal PbS QDs dispersed in toluene with an average diameter of approximately 5.1 nm. The broadband optical absorption is measured by a Fourier transform infrared (FTIR) spectrometer (Bomem DA8). For the ultrafast pump-probe spectroscopy, the colloidal PbS QDs are maintained in a 3-mm cell contained in the toluene liquid with two optically-transparent MgO windows, and the samples are actively stirred using a magnetic stirrer to ensure that photo-charging does not occur during the measurements (Fig. 1b)^50^. Using a 250 kHz Ti-sapphire regenerative amplifier (Coherent RegA 9050), the samples are excited by 50 fs pulses with a pump-photon energy *E*_pump_ of 1.55 eV and its second harmonic *E*_pump_ of 3.10 eV for investigating the MEG photo-dynamics. A fraction of the amplifier output is used as a probe pulse with photon energy *E*_probe_ of 0.93 eV for the lowest *E*_x_ and 0.87 eV for the *E*_xx_. Both probe pulses are delivered from wavelength-tunable optical parametric amplifier (Coherent OPA 9850).

## Author Contributions

H.C. and Y.H.L. developed the original experimental ideas. Y.C. and S.S. performed the ultrafast pump-probe measurements. Y.C. and S.C.L. prepared the colloidal QD samples and analyzed the data. The manuscript was written through contributions of all authors.
